# Hybrid Surgical Approach for Acute Limb Ischemia: A Case Report and Management Algorithm

**DOI:** 10.7759/cureus.103606

**Published:** 2026-02-14

**Authors:** Constantine N Antonopoulos, Christos F Pitros, Andreas Panagiotopoulos, Andreas M Lazaris

**Affiliations:** 1 First Department of Vascular Surgery, Attikon University Hospital, National and Kapodistrian University of Athens, Athens, GRC

**Keywords:** acute limb ischemia, catheter-directed thrombolysis, hybrid revascularization, popliteal artery aneurysm, threatened limb

## Abstract

Acute limb ischemia (ALI) is a time-critical vascular emergency associated with high morbidity and amputation risk if treatment is delayed. Popliteal artery aneurysms (PAAs), though less common, represent an important and often underrecognized cause. We report a 67-year-old man who presented with sudden-onset severe leg pain, pallor, sensory loss, and motor deficit, consistent with Rutherford IIb ALI. Computed tomography angiography revealed a nearly thrombosed PAA with absent distal run-off. The patient underwent hybrid revascularization combining open thrombo-embolectomy with prosthetic interposition grafting, intra- and postoperative catheter-directed thrombolysis, and prophylactic four-compartment fasciotomies. Postoperatively, distal perfusion improved significantly, sensation fully recovered, motor function was nearly restored, and independent ambulation was achieved. This case highlights extensive distal embolization with absent tibial run-off, a condition in which isolated embolectomy is often inadequate. It illustrates the effectiveness of a hybrid surgical-endovascular strategy for limb salvage and underscores the importance of rapid imaging, urgent revascularization, adjunctive thrombolysis, and early fasciotomy. These principles are summarized in a practical management algorithm.

## Introduction

Arterial embolism remains a common cause of acute limb ischemia (ALI) and is associated with significant morbidity and limb loss if not recognized and treated promptly [1,2]. Although cardiac emboli account for the majority of cases, peripheral arterial aneurysms represent an important non-cardiac source of distal embolization and acute ischemic presentations.

Popliteal artery aneurysms (PAAs) are the most frequently encountered peripheral arterial aneurysms and typically present with thrombosis or embolization rather than rupture [3-5]. ALI related to PAAs continues to pose a significant clinical challenge, often complicated by distal thromboembolism and poor runoff, which adversely affects revascularization outcomes and limb salvage [3,6]. Contemporary series report persistent morbidity despite advances in surgical technique, emphasizing the importance of timely diagnosis and appropriate intervention [6,7].

Management of ALI secondary to PAAs must address both the restoration of distal perfusion and definitive exclusion of the aneurysm. While open surgical repair remains the standard treatment, outcomes may be limited in the setting of extensive distal embolization. Increasing experience supports the selective use of hybrid strategies combining surgical revascularization with adjunctive endovascular techniques to improve distal runoff and optimize limb salvage in selected patients presenting with acute ischemia due to PAAs [6,7].

Decision-making in patients with Rutherford IIb [8] ALI and absent distal runoff remains insufficiently defined, particularly regarding the optimal integration and timing of adjunctive endovascular techniques alongside open repair. This uncertainty represents an important clinical gap that the present case seeks to address.

## Case presentation

A 67-year-old male patient presented to the Emergency Department with a sudden onset of severe left lower limb pain and dysfunction that had begun only a few hours before arrival. On clinical examination, the left lower limb appeared pale and cold with no capillary refill. Distal sensation was reduced, foot motor function was impaired, distal pulses were absent, and there was marked calf tenderness. His past medical history included type 2 diabetes mellitus treated with metformin.

**Figure 1:**
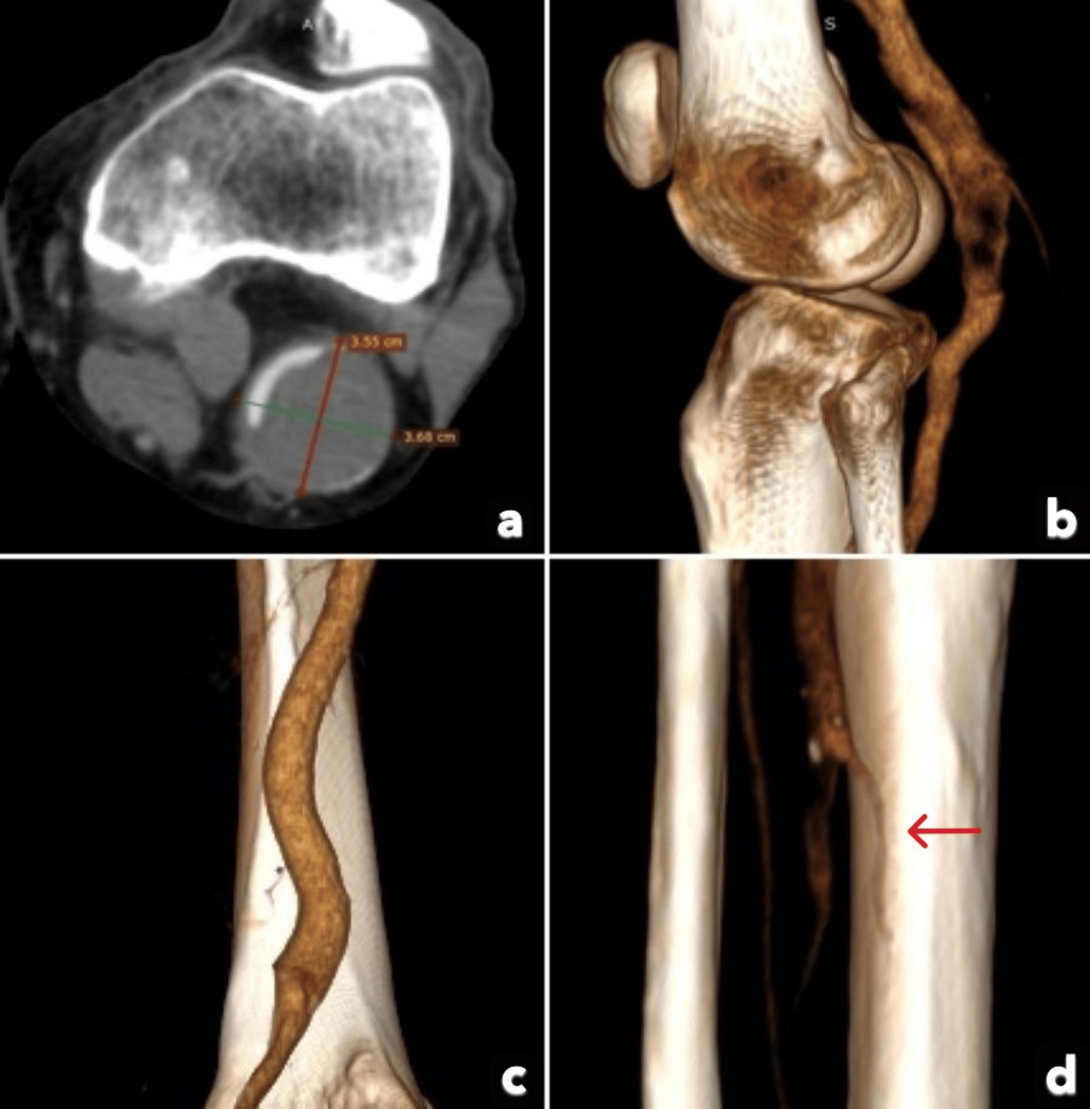
Preoperative CTA showing almost complete thrombosis of the popliteal artery aneurysm (PAA) and no distal vessel run-off Absence of distal vessel run-off due to the thrombosed PAA (red arrow).

Laboratory studies showed a hematocrit of 33% (reference value: 40-52% (male)), white blood cell count of 22,800/μL (reference value: 4,000-12,000/μL), and creatine kinase of 2,383 U/L (reference value: 30-200 U/L). Computed Tomography Angiography (CTA) imaging (Figures 1a-1d) revealed an almost completely thrombosed PAA measuring 3.6 × 3.7 cm, along with occlusion of the mid-calf anterior tibial, posterior tibial, and peroneal arteries, resulting in the absence of distal vessel run-off. 

Assessment of the contralateral limb was performed as a part of the imaging workup and demonstrated no evidence of aneurysmal disease.

**Figure 2:**
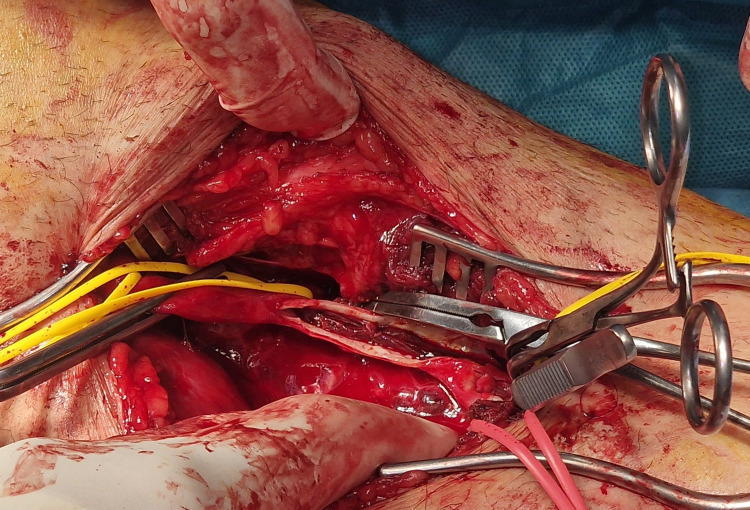
Thrombus found after opening the below-knee popliteal artery with trifurcation

The patient underwent emergency surgery that involved open exposure of the above-knee and below-knee popliteal artery with trifurcation (Figure 2), proximal and distal thrombo-embolectomy of the calf vessels using a Fogarty catheter, and interposition of a 6-mm expanded polytetrafluoroethylene (ePTFE) graft into the thrombosed PAA.

A prosthetic graft was chosen to reduce the operative time and allow the fastest possible restoration of perfusion given the severity of ischemia. Intraoperative catheter-directed thrombolysis of the distal vessels was performed, followed by the percutaneous placement of a distal arterial catheter for 24-48 hours of thrombolysis via a common femoral artery sheath.

**Figure 3:**
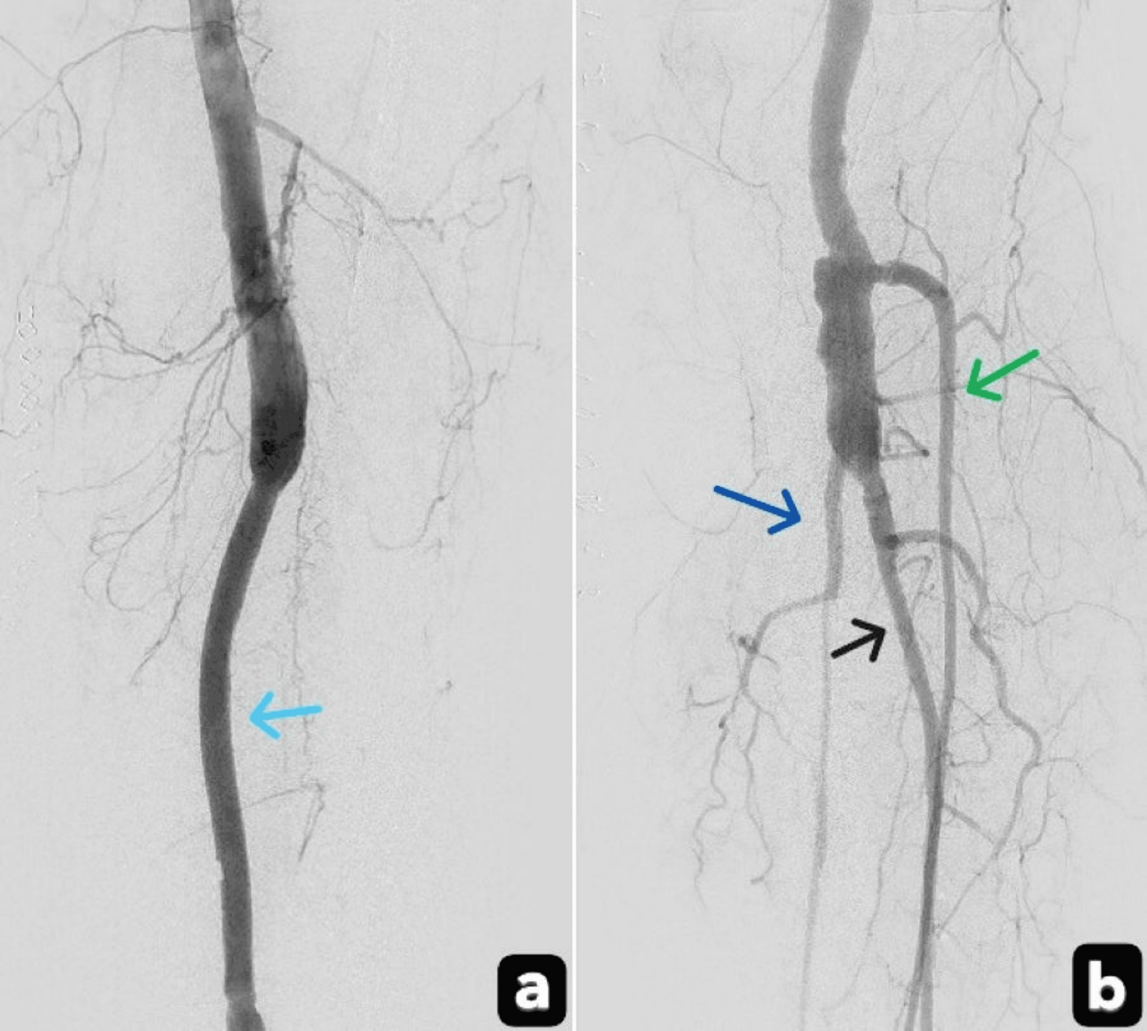
Digital subtraction angiography demonstrated enhanced distal runoff Patent popliteal artery interposition graft (Light blue arrow); Patent anterior tibial artery (green arrow); Patent posterior tibial artery (Blue arrow); Patent peroneal artery (Black arrow).

To prevent reperfusion-induced compartment syndrome, two-incision (medial and lateral), four-compartment fasciotomies were also performed. Postoperatively, the patient was admitted to the ICU for close monitoring of catheter-directed thrombolysis, with serial neurological assessments and laboratory testing. Digital subtraction angiography (Figures 3a, 3b) demonstrated improved distal runoff.

**Figure 4:**
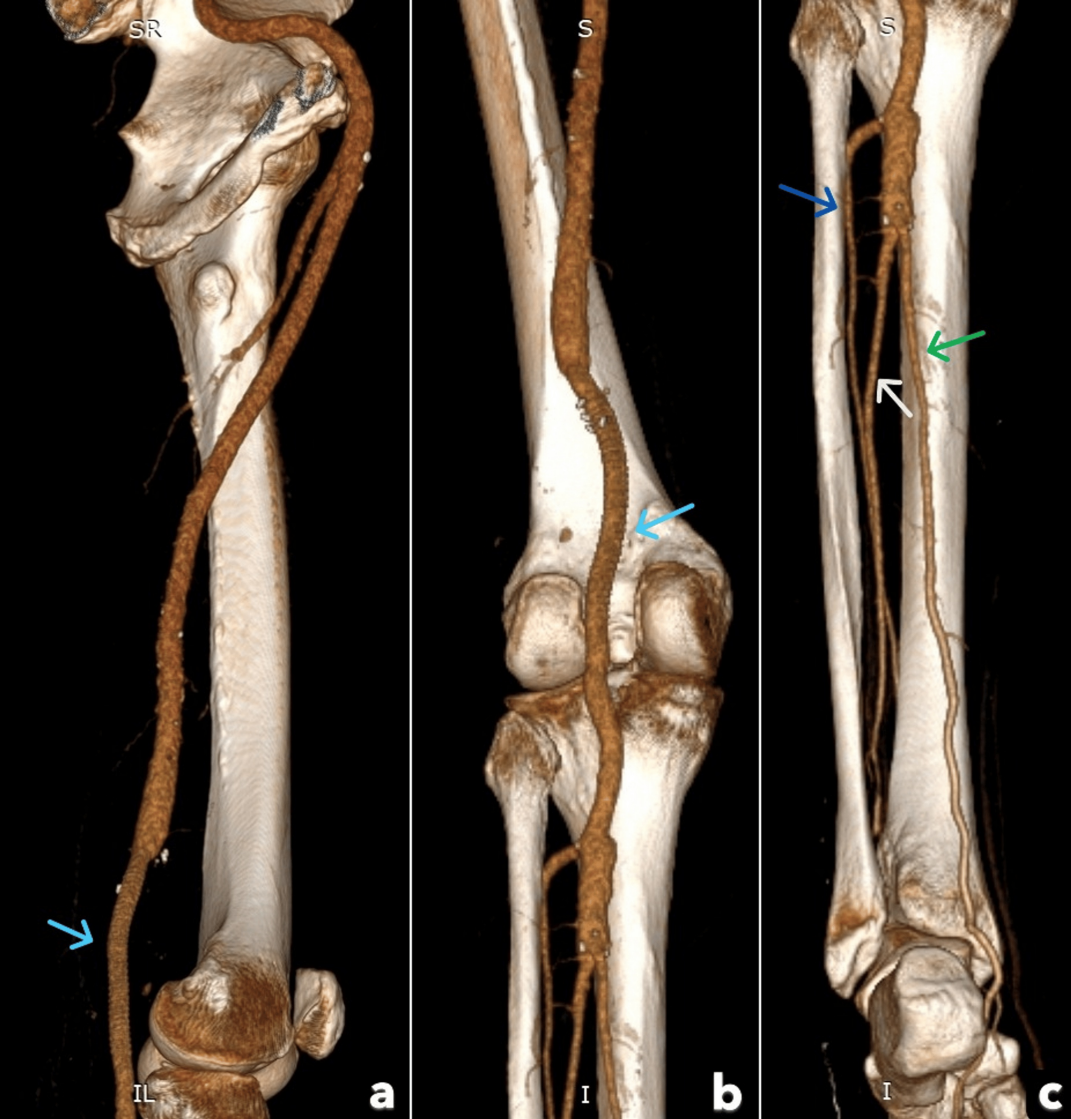
The post-operative CTA showed graft patency with restored distal vessel run-off Patent popliteal artery interposition graft (Light blue arrow); Patent anterior tibial artery (Blue arrow); Patent posterior tibial artery (Green arrow); Patent peroneal artery (White arrow).

Clinically, the patient recovered full sensory function and most motor activity, with only mild residual weakness in dorsiflexion of the forefoot due to deep peroneal nerve paresis. He regained independent ambulation with temporary use of assistance. Postoperative CTA confirmed graft patency with restored distal vessel run-off (Figures 4a-4c).

The management of ALI caused by a thrombosed PAA in this patient is summarized in Table 1.

**Table 1:** Management of acute limb ischemia due to thrombosed popliteal artery aneurysm (PAA) CTA: Computed Tomography Angiography.

Steps	Action	Purpose
Emergency Diagnosis	CTA	Confirm PAA thrombosis and distal vessel run-off status
Surgical Approach	Open surgical bypass and embolectomy	Restore inflow and outflow
Adjunctive Therapy	Catheter-directed thrombolysis (24–48 hours)	Improve tibial vessel run-off
Prevention of lower limb muscle necrosis	Prophylactic 4-compartment fasciotomies	Prevent compartment syndrome
ICU Monitoring	Continue thrombolysis, continuous limb assessment	Improve run-off and check for early detection of complications
Rehabilitation	Physiotherapy and surveillance	Restore lower limb function and monitor graft patency

Closure of the fasciotomy wounds was facilitated using the shoelace technique. The postoperative course was uneventful, without major local or systemic complications.

## Discussion

**Figure 5:**
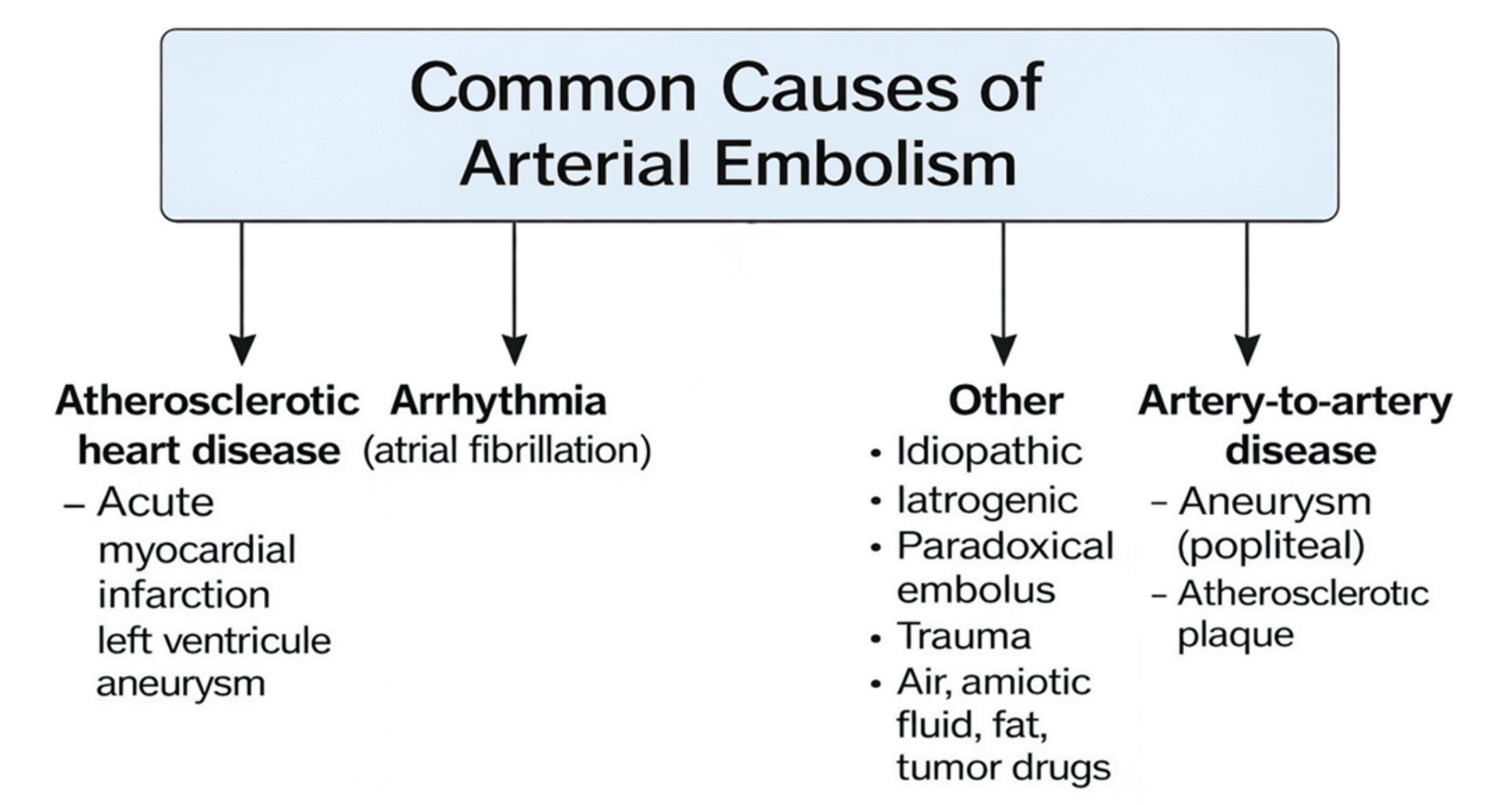
Common causes of arterial embolism Image credit: Created by Antonopoulos CN using Microsoft PowerPoint (Microsoft Corp., Redmond, WA, USA).

The management of thrombosed PAAs presenting with ALI is among the most complex challenges in vascular surgery, with high risks of morbidity and amputation despite advances in imaging, operative techniques, and adjunctive therapies [3,6,7]. The most common causes are depicted in Figure 5.

The present case reflects evolving principles of care that have emerged from large contemporary clinical series [3,6,7]. Rapid intervention is essential, as delay in revascularization is associated with significantly worse limb salvage outcomes [3,8].

The Rutherford classification remains a practical framework for determining urgency and guiding management in ALI. Patients with immediately threatened limbs (category IIb), such as in this case, typically present with rest pain, sensory loss, mild motor weakness, delayed or absent capillary refill, absent arterial but preserved venous Doppler signals, and require urgent revascularization to prevent irreversible ischemic injury [8]. Systemic anticoagulation with heparin is a standard initial measure; however, in the presence of neurological deficit, anticoagulation alone is insufficient and definitive revascularization is mandatory [3,8].

CTA has become the imaging modality of choice in ALI, allowing rapid and accurate assessment of aneurysm morphology, thrombus burden, distal tibial runoff, and contralateral arterial disease. This facilitates timely operative planning and selection of open, endovascular, or hybrid strategies in the acute setting [2,8].

Although endovascular repair is well established in elective PAA management, its role in ALI remains limited. Emergency stent grafting has been associated with lower primary patency rates and higher early reintervention compared with open surgery [6]. Consequently, open surgical repair, often combined with adjunctive catheter-directed thrombolysis, remains the preferred approach in urgent cases, as it allows restoration of both inflow and distal outflow and maximizes limb salvage [6,7].

Conduit selection is a critical determinant of outcome. Autologous great saphenous vein remains the conduit of choice for below-knee bypasses, offering superior long-term patency and limb preservation. Prosthetic grafts such as ePTFE may be acceptable for above-knee reconstructions or when ischemia time must be minimized [4,9]. In the present case, the choice of a prosthetic conduit prioritized immediate reperfusion in a severely threatened limb, accepting the potential tradeoff with long-term durability. Endo-aneurysmorrhaphy with short interposition grafting has also demonstrated satisfactory outcomes in selected urgent cases, with results comparable to vein bypass in experienced centers [9,10]. The use of prosthetic material inevitably raises concern regarding postoperative infection, a complication that can severely compromise outcomes after vascular reconstruction. In recent years, interest has grown in laboratory biomarkers that might facilitate early identification of septic complications. Among these, butyrylcholinesterase has shown promise in other surgical disciplines [11]. However, its predictive value in ALI and urgent vascular procedures remains insufficiently established.

Adjunctive catheter-directed thrombolysis represents a cornerstone of hybrid management in thrombosed PAAs presenting with ALI. While surgical repair restores inflow, distal embolization frequently persists within the tibial vessels. Intraoperative thrombolysis, followed by short-term postoperative infusion, improves tibial runoff and distal perfusion [6,7]. The extent of tibial vessel recanalization is a key determinant of limb salvage, with a three-vessel runoff strongly associated with favorable outcomes and absence of runoff linked to amputation [7]. However, thrombolysis carries inherent risks, including major bleeding and hemorrhagic stroke, necessitating careful patient selection and close monitoring [4]. In this patient, risk mitigation relied on intensive care monitoring with repeated neurological and vascular assessments, routine laboratory follow-up, and strict surveillance of the access site.

Reperfusion following prolonged ischemia also carries a substantial risk of compartment syndrome. Although the optimal timing of fasciotomy remains controversial, in this patient decompression was undertaken concurrently with revascularization to minimize the risk of reperfusion injury. Prophylactic fasciotomy has been shown to reduce limb loss in patients undergoing revascularization for ALI, particularly in the presence of prolonged ischemia, elevated creatine kinase levels, marked limb swelling, or extensive thrombus burden [3,8]. Four-compartment fasciotomy is effective in preventing irreversible neuromuscular injury. Adjunctive wound management techniques, including elastic shoelace suturing, negative pressure wound therapy, and split-thickness skin grafting, facilitate delayed closure when primary closure is not feasible [2,12].

Emergent endovascular stent grafting in ALI has produced inferior outcomes compared with open or hybrid repair. Satam et al. reported higher rates of amputation, reintervention, and perioperative mortality following emergent endovascular treatment of thrombosed PAAs [6]. As such, while endovascular exclusion remains appropriate in selected elective patients, open or hybrid repair offers superior outcomes in the setting of ALI.

Predictors of poor prognosis include absent tibial runoff, delayed presentation beyond six to 12 hours, delayed fasciotomy, significant comorbidities such as renal dysfunction, diabetes, or coronary artery disease, and the use of prosthetic grafts for distal anastomosis [4]. Rigorous postoperative surveillance with duplex ultrasonography is essential, as most graft failures occur within the first 18 months. Long-term success further depends on cardiovascular risk factor modification and structured follow-up [4].

Long-term outcomes following open repair are favorable when fundamental principles are applied. Dorigo et al. reported a 13-year limb preservation rate of 86% and amputation-free survival exceeding 80% at five years [4]. Hybrid strategies combining surgical repair with catheter-directed thrombolysis have emerged as the contemporary standard for thrombosed PAAs presenting with ALI, offering the best opportunity for durable patency, limb salvage, and functional recovery. The near-complete sensory and motor recovery observed in this case underscores the effectiveness of timely, aggressive, and evidence-based multimodal management.

**Figure 6:**
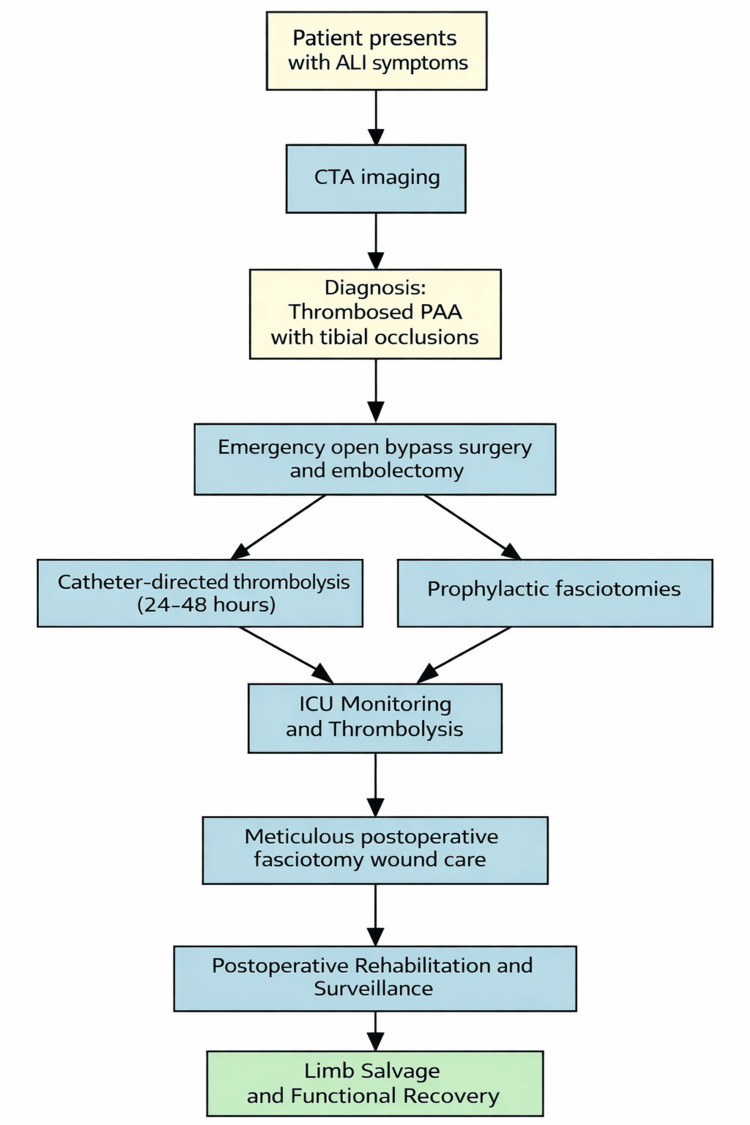
Proposed algorithm for patients with ALI due to PAAs with distal vessel thromboembolism ALI: Acute limb ischemia; PAA: Popliteal artery aneurysms. Image credit: Created by Antonopoulos CN using Microsoft PowerPoint (Microsoft Corp., Redmond, WA, USA).

For patients presenting with thrombosed PAAs complicated by ALI, we propose a pragmatic management algorithm to guide urgent decision-making and treatment selection in real-world practice (Figure 6).

## Conclusions

A hybrid approach combining open surgical revascularization with adjunctive catheter-directed thrombolysis is an effective option for limb salvage in patients presenting with thrombosed PAAs and ALI. Favorable outcomes in such complex presentations depend heavily on early patient presentation, rapid access to advanced imaging, and the availability of multidisciplinary expertise. These factors may limit the generalizability of this approach in lower-resource settings. Prophylactic fasciotomy to reduce the risk of reperfusion injury and structured postoperative rehabilitation are important components of care. When applied together, these measures can result in meaningful functional recovery, even in severe cases of ALI.
